# Snorkel Stenting During Transcatheter Aortic Valve Implantation: A Single-Center Study

**DOI:** 10.3390/medicina61091679

**Published:** 2025-09-16

**Authors:** Massimo Baudo, Serge Sicouri, Yoshiyuki Yamashita, Dimitrios E. Magouliotis, Francesco Cabrucci, Sarah M. Carnila, Scott M. Goldman, Eric M. Gnall, William A. Gray, Sandro Gelsomino, Basel Ramlawi

**Affiliations:** 1 Department of Cardiac Surgery Research, Lankenau Institute for Medical Research, Main Line Health, Wynnewood, PA 19096, USA; sicouris@mlhs.org (S.S.); yoshiyuki.yamashita.md@gmail.com (Y.Y.); magouliotisd@mlhs.org (D.E.M.); francesco.cabrucci.6@gmail.com (F.C.); sarahmaggie14@gmail.com (S.M.C.); grayw@mlhs.org (W.A.G.); ramlawib@mlhs.org (B.R.); 2 Department of Cardiac Surgery, Lankenau Heart Institute, Main Line Health, Wynnewood, PA 19096, USA; goldmans@mlhs.org; 3 Department of Interventional Cardiology, Lankenau Heart Institute, Main Line Health, Wynnewood, PA 19096, USA; gnalle@mlhs.org; 4 Cardiovascular Research Institute Maastricht-CARIM, Maastricht University Medical Centre, 6229HX Maastricht, The Netherlands; sandro.gelsomino@maastrichtuniversity.nl

**Keywords:** tavi, transcatheter, aortic valve, coronary obstruction, snorkel stenting, chimney stenting

## Abstract

*Background and Objectives*: Coronary obstruction (CO) is a potentially fatal complication that can occur during transcatheter aortic valve implantation (TAVI). The snorkel stenting technique was developed to mitigate the risk of CO. The aim of the current study was to evaluate the safety and clinical outcomes of snorkel stenting in patients at high risk of developing CO. *Materials and Methods*: All patients undergoing TAVI with snorkel stenting between January 2018 and December 2023 were retrospectively included. The primary outcome was the evaluation of perioperative CO and myocardial infarction (MI) at follow-up. *Results*: Between 2018 and 2023, a total of 1476 TAVI procedures were conducted, with 21 patients undergoing snorkel stenting for CO prevention. Median STS was 5.0 [IQR 3.0–8.0]. Four patients (19.0%) had a history of prior percutaneous coronary intervention, and 85.7% (*n* = 18) had a degenerated bioprosthesis. Preoperative multidetector CT revealed a mean sinus of Valsalva diameter of 24.6 ± 6.8 mm and a mean annulus area of 402.0 ± 59.7 mm^2^. The mean heights of the left and right coronary ostia were 7.9 ± 3.3 mm and 10.6 ± 5.5 mm, respectively. Nine patients (42.9%) presented with severe aortic regurgitation. Snorkel stenting was performed on the left main coronary in nine cases, on the right coronary in 10 cases, and on both the left main and right coronary arteries in two cases. No procedural complications were observed. Postoperatively, there were no MIs; one patient suffered from a disabling stroke, and another patient required a new pacemaker implant. Thirty-day mortality rate was 0%. At a median follow-up of 1.25 years (IQR: 1.05–3.05), there were five rehospitalizations for heart failure, no aortic valve reinterventions, one disabling stroke (sub-acute), and one MI. Overall, four patients died, including two cardiovascular-related deaths. *Conclusions*: Snorkel stenting should be considered for selected high- or prohibitive-surgical-risk patients undergoing TAVI who are at increased risk of developing CO. This technique showed favorable procedural outcomes for preventing CO occurring during or in the perioperative period of TAVI procedures. The abstract of the present study was accepted for oral presentation at the New York Valves on 26 June 2025.

## 1. Introduction

Coronary obstruction (CO) is a rare yet serious adverse event that can occur following transcatheter aortic valve implantation (TAVI). It is characterized by newly detected partial or total blockage of an epicardial coronary artery, identified through angiography or echocardiography [1]. This obstruction may result from various mechanisms, including displacement of the native valve leaflets over the coronary ostia, occlusion caused by the transcatheter heart valve (THV) itself, sequestration of the sinotubular junction, or embolic material lodging within the coronary arteries, each leading to myocardial ischemia [1].

Several clinical and anatomical factors have been linked to an increased risk of CO following TAVI in native aortic valves [2]. Clinically, advanced age, lack of prior coronary artery bypass grafting, and use of early-generation balloon-expandable valves have been associated with higher CO risk. Anatomically, a sinus of Valsalva height less than 30 mm and a left main coronary artery height under 12 mm have been recognized as key predictors [3,4]. More recently, additional structural risk factors have been identified. These include narrowed sinus width, as well as a post-TAVI residual sinus width of less than 5 mm. Another important indicator is when the coronary cusp height exceeds the coronary height. Additionally, native leaflet bulk, estimated by calcium volume, has been implicated; a calcium volume exceeding 600 mm^3^ has been shown to be a useful threshold for predicting CO risk [5].

**Figure 1:**
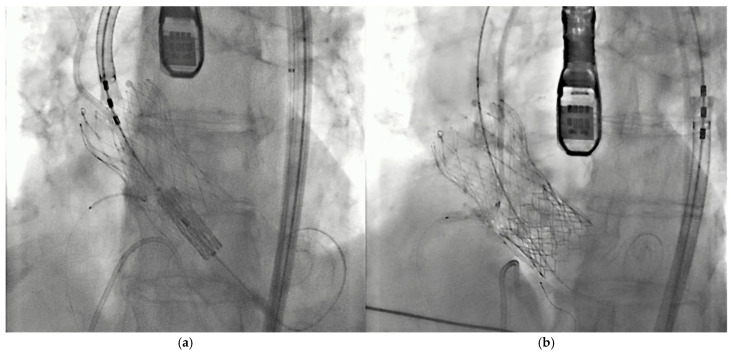
Snorkel stenting during TAVI in one of the study patients. In this particular case, a valve-in-valve procedure was performed, involving the implantation of a balloon-expandable valve within a previously placed self-expanding valve. (**a**) The right coronary artery is pre-wired prior to THV deployment to allow for stenting. (**b**) The coronary stent is then positioned and deployed following THV implantation.

Various procedural strategies have been developed to help minimize the likelihood of CO following TAVI. The most important include the BASILICA technique (Bioprosthetic or Native Aortic Scallop Intentional Laceration to Prevent Iatrogenic Coronary Artery Obstruction) [6] and the Chimney/Snorkel stenting technique [7]. The BASILICA procedure works by lacerating the targeted aortic leaflet to create a triangular opening, allowing blood to flow into the sinuses and reach the coronary arteries. However, its effectiveness can be reduced if the leaflets are heavily calcified. Additionally, this technique may be ineffective in cases involving substantial commissural misalignment in a degenerated bioprosthetic valve. On the other hand, the snorkel stenting involves pre-positioning a guidewire and stent in the at-risk coronary artery before valve implantation (Figure 1a). If an obstruction occurs after valve deployment, the stent is pulled back to cover the coronary ostium. Part of the stent then protrudes into the aorta to prevent blockage by the native leaflet (Figure 1b).

The objective of this study was to evaluate clinical outcomes of patients who underwent TAVI with snorkel stenting for coronary protection in a single medical center.

## 2. Materials and Methods

Between January 2018 and December 2023, a cohort of patients who underwent TAVI with snorkel stenting for coronary protection at the Lankenau Heart Institute, Wynnewood, PA, USA, was retrospectively analyzed. In all patients, snorkel stenting involved both guidewire positioning and stent deployment; none underwent guidewire positioning alone. Postprocedural coronary flow was assessed through the TIMI flow grade [8]. The objective of this investigation was to evaluate clinical outcomes in this specific patient population, and no other inclusion/exclusion criteria have been applied besides undergoing snorkel stenting during TAVI.

The study protocol received approval from the Institutional Review Board of Main Line Health Hospitals (IRB 45CFR164.512), and a waiver of informed consent was granted, given the retrospective design.

Patient clinical, procedural, and follow-up information was obtained from electronic health records. All individuals had undergone pre-TAVI imaging, including transthoracic and/or transesophageal echocardiography, along with multi-detector computed tomography (MDCT) to guide procedural planning.

The primary endpoint was to assess the safety and efficacy of snorkel stenting in patients at an elevated risk of CO, with a specific emphasis on the incidence of CO and myocardial infarction (MI).

### 2.1. Snorkel Stenting Approach

Based on previously reported techniques, snorkel stenting as a protection strategy begins with the identification of patients at high risk of CO [9,10]. The parameters considered to evaluate the CO risk were low coronary height, narrow and/or short sinuses, cusp height > coronary height, or in the context of valve-in-valve TAVI (stentless valve, stented valve with external leaflets, implantation depth).

In these cases, a coronary guidewire was advanced into the target coronary artery, and an appropriately sized drug-eluting stent was positioned across the coronary ostium inside the coronary artery, ready for deployment. The THV was then implanted in the standard fashion, followed by any necessary post-dilatation to optimize expansion and minimize paravalvular leak. Only after the valve had been fully deployed and post-dilatation completed was the stent pulled back into the aorta and deployed, with slight protrusion into the aortic root to achieve the characteristic “snorkel” configuration.

This sequence was performed to reduce the risk of stent crushing by the expanding valve frame. Finally, the stent was post-dilated to ensure complete expansion and apposition, and the outcome was confirmed by angiography with TIMI flow assessment to verify preserved coronary perfusion and satisfactory valve function with echocardiography.

### 2.2. Definitions

Clinical data collection adhered to the standardized terminology and variable definitions established by the STS/ACC Transcatheter Valve Therapy (TVT) Registry [11]. Renal function was assessed using the estimated glomerular filtration rate (eGFR), calculated according to the 2021 CKD-EPI creatinine-based formula [12].

Composite outcomes of technical success, device success, and early safety were evaluated in accordance with the criteria defined by the Valve Academic Research Consortium-3 (VARC-3) [1].

### 2.3. Statistical Methods

Categorical variables were summarized using counts and percentages. To determine the distribution of continuous variables, the Kolmogorov–Smirnov test was applied. Variables with a normal distribution were presented as mean ± standard deviation, while those not normally distributed were described using median and interquartile range (IQR).

Data processing and analysis were performed using R software (version 4.4.2) via the RStudio environment (R Project for Statistical Computing, Vienna, Austria).

In accordance with institutional policies and upon reasonable request, the dataset underlying the study’s findings can be made available. The study was conducted and reported in line with the STROBE (STrengthening the Reporting of Observational Studies in Epidemiology) guidelines for observational studies [13].

## 3. Results

Between 2018 and 2023, our center performed 1476 TAVI procedures, of which 21 involved snorkel stenting. Over 70% (*n* = 15) of the procedures were performed within the last three years, whereas the initial attempts were only sporadically carried out in earlier years. Mean age was 81.3 ± 6.1, and 16 (76.2%) were female. Most patients were symptomatic with a median STS-PROM of 5.0 [IQR: 3.0–8.0]. Preoperative demographics are summarized in Table 1.

At MDCT scan, patients generally presented a small root conformation with a mean annulus diameter of 21.2 ± 2.5 mm, a mean area of 402.0 ± 59.7 mm^2^, and a mean perimeter of 71.7 ± 5.2 mm. Left and right mean coronary height were 7.9 ± 3.3 mm and 10.6 ± 5.5 mm, respectively (Table 2). No information on valve calcification could be provided, as most cases involved valve-in-valve TAVI. The presence of a prior valve, many of which contain metal, interferes with calcium score calculation, rendering it unreliable.

At echocardiography, nine (42.9%) patients suffered from severe aortic regurgitation. Eighteen (85.7%) patients were referred for a degenerated bioprosthetic valve. The most frequent one was Trifecta (Abbott Inc., Chicago, IL, USA) in a third of the cases (*n*= 7, 33.3%), followed by MitroFlow (Sorin Medical, Milan, Italy) (*n* = 3, 14.3%), Freestyle (Medtronic Inc., Minneapolis, MN, USA) (*n* = 2, 9.5%), and CoreValve (Medtronic Inc., Minneapolis, MN, USA) (*n* = 2, 9.5%), as shown in Table 2.

Intraoperatively, all but one patient underwent the procedure via a transfemoral approach (*n* = 20, 95.2%), while the remaining patient was treated using transaxillary access. A self-expanding valve was deployed in 13 patients (61.9%), while a balloon-expandable valve was used in the remaining 8 patients (38.1%). Snorkel stenting of the left main and right coronary arteries was evenly distributed, as detailed in Table 3. All patients received a stent as planned, with no problems with stent deployment or opening.

There was only one major perioperative complication: a case of disabling stroke that occurred three days after the procedure. However, there were no episodes of CO or MI. Four patients (19.0%) developed a new left bundle branch block, one patient (4.8%) required a new permanent pacemaker, and one patient (4.8%) received a postoperative blood transfusion. At discharge, echocardiography demonstrated favorable THV function, with only two patients (9.8%) exhibiting mild paravalvular leaks. A comprehensive summary of perioperative outcomes is provided in Table 3.

At discharge, 15 patients (71.4%) were prescribed dual antiplatelet therapy (DAPT), 5 patients (23.8%) received a combination of DAPT and anticoagulation, and 1 patient (4.8%) was discharged on single antiplatelet therapy with anticoagulation.

Technical and device success rates, according to VARC-3 criteria, were 100% and 81.0% (*n* = 17), respectively. Early safety at 30 days was achieved in 90.5% of patients (*n* = 19) (Table 4).

At a median follow-up of 1.25 years [IQR: 1.05–3.05], overall survival was 81.0% (four all-cause deaths), with two cardiovascular-related deaths (9.5%). One patient died due to a MI. The patient had a history of prior CABG surgery on the left anterior descending and obtuse marginal arteries seven years before, and a stent was placed in the right coronary artery. The exact etiology of the right-sided MI remained undetermined but was probably related to the newly implanted stent. The other patient died due to a pulmonary embolism. Echocardiographic assessments at 1 month and 1 year demonstrated favorable THV hemodynamics, with only three cases (15.8%) of mild paravalvular leak. Another patient suffered from a disabling stroke after discharge within 30 days of the procedure (sub-acute). All follow-up outcomes are detailed in Table 4.

## 4. Discussion

This study assessed the outcomes of snorkel stenting in patients undergoing TAVI at high risk of developing CO. The key findings are summarized as follows: (1) The majority of patients had a previously implanted aortic valve. (2) Only one patient suffered from a major perioperative complication (stroke), but none suffered CO or MI. At follow-up, there was only one case (4.8%) of MI and one sub-acute disabling stroke (4.8%). (3) Four patients (19%) developed a new left bundle branch block, and one (4.8%) required permanent pacemaker implantation. (4) At discharge, only two patients (9.5%) had a mild paravalvular leak. (5) Overall, the VARC-3-defined clinical outcomes were highly encouraging.

CO represents a serious and potentially fatal complication of TAVI, with reported in-hospital mortality rates reaching as high as 50% [14]. This risk is particularly pronounced in valve-in-valve procedures [15]. Pre-procedural MDCT imaging plays a critical role in identifying patients who are at an elevated risk [16]. In this regard, parameters such as the valve-to-coronary (VTC) distance, the valve-to-sinotubular junction (VTSTJ) distance, and the valve-to-aortic wall (VTA) distance are fundamental and could guide procedural strategies [17].

For these high-risk patients, various preventive strategies have been developed, most notably techniques such as chimney/snorkel stenting and the BASILICA procedure. Other techniques are currently being investigated:

− To enhance leaflet separation during the BASILICA procedure, a modified technique called balloon-augmented (BA)-BASILICA has been developed. This variation follows the traditional BASILICA workflow but introduces a crucial step: just before the leaflet is lacerated, a 4 to 5 mm balloon is inflated within the targeted cusp to facilitate a wider splay [18]. The preliminary data documented one case each of CO (6%), stroke (6%), and life-threatening bleeding (6%), along with two cases of major vascular complications (13%). No 30-day mortality was reported. The more established nature of snorkel stenting compared with BA-BASILICA may account for these differences and highlights the need for further outcome improvements.
− Developed by Pi-Cardia (Rehovot, Israel), the ShortCut device is engineered to modify valve leaflets [19]. It employs a dual-arm mechanism at its tip; one arm positions the device, while the other performs the split, allowing for precise scissor-like cutting once optimal alignment is reached. Similarly to our study, no cases of CO, moderate/severe aortic regurgitation, or 30-day mortality were observed, with a median transvalvular gradient of 10 mmHg. No stroke occurred, and no new pacemaker implantations were required; one major vascular complication was reported.
− The CLEVE consists of excising a leaflet from a surgically implanted valve, particularly in cases where its presence poses a risk of obstructing the coronary ostia or the left ventricular outflow tract [20]. The process begins by using a 0.014-inch guidewire, stripped to expose the metallic core, through which 70 watts of “cut” mode electrosurgical energy is applied to perforate the target leaflet. After the wire traverses into the ventricular chamber, the leaflet is progressively expanded using balloon dilatation, first with a 4.0 × 20 mm balloon, then with a larger 12.0 × 40 mm balloon. Once the leaflet is adequately modified, a THV is deployed using routine implantation methods. Among the eight patients analyzed, there was one case of CO and one 30-day mortality. No paravalvular leak or stroke was observed, and the mean transvalvular gradient was 7.8 mmHg.
− Building upon the principles of the BASILICA technique, CATHEDRAL (CATHeter Electrosurgical Debulking and RemovAL) introduces a refined method for leaflet removal [21]. A modified “Flying V” wire configuration is used, not for laceration, but to grasp and manipulate the targeted leaflet. Once the leaflet is positioned, a snare is deployed at its base, delivering electrosurgical energy to detach it. The freed leaflet is then guided via the Flying V into a larger sheath for extraction and removal from the body.
− The LLAMACORN (Leaflet Laceration And Midline Approximation to Create an Orifice Resembling Native) technique is designed to reconfigure the bioprosthetic valve opening, restoring a more anatomically native shape [22]. It is especially useful in cases of pronounced commissural misalignment or distorted, non-circular valve orifices, which can hinder proper transcatheter valve expansion or performance. In all five cases, TAVI was performed successfully without complications. None developed CO, conduction disturbances requiring pacemaker implantation, stroke, or vascular complications. All patients were discharged home and remained alive at 30 days. The mean transvalvular gradient was 11.6 mmHg, with only one case of mild paravalvular leak.
− UNICORN (Undermining Iatrogenic Coronary Obstruction With Radiofrequency Needle) is an emergent rescue technique developed to restore coronary perfusion after a coronary obstruction has occurred during TAVI [23]. It is particularly valuable in cases where no preemptive protective measures, such as coronary wiring or stenting, were implemented prior to the obstruction.

Unlike BASILICA and snorkel stenting, which are more established techniques, these alternative procedures remain investigational and require validation through larger studies with extended follow-ups.

Snorkel stenting has been associated with a lower risk of coronary obstruction compared to coronary wiring alone. In this regard, the CORPROTAVR (CORonary PROtection during TAVR) was a retrospective, multicenter, international registry comparing the two approaches in patients undergoing TAVI who were considered at high risk of coronary obstruction [24]. Among patients who received coronary stents, two definite cases of stent thrombosis were observed (0.9%), both following valve-in-valve TAVI procedures. In contrast, among those who did not receive stents, four delayed CO were reported (4.3%). After three years of follow-up, cardiac mortality was observed in 7.8% of patients treated with stents compared to 15.7% in those without stents. Although the adjusted hazard ratio favored stenting (HR 0.42; 95% CI: 0.14–1.28), the difference did not reach statistical significance (*p* = 0.13).

The multicenter Chimney registry evaluated the safety and effectiveness of chimney stenting as a bailout technique to manage CO during TAVI [25]. The procedure was used either in response to an established CO (41.6%) or to prevent an impending CO (58.3%). Procedural and in-hospital mortality were low, with one and two cases, respectively. However, MI, cardiogenic shock, and resuscitation occurred significantly more often in patients with established CO than in those with impending CO. The lack of upfront coronary protection was identified as the only independent predictor of the composite outcome of death, cardiogenic shock, or MI. Over a median follow-up of 612 days, two instances of stent failure were recorded. Overall, chimney stenting was found to be a viable bailout strategy for CO, with better outcomes seen in patients who received proactive coronary protection.

Consistent with findings from the Chimney Registry, the present study supports the value of proactive coronary protection in reducing the incidence of CO among high-risk patients. Although the sample size was limited, no perioperative cases of CO or MI were observed, and only a single MI occurred during follow-up. VARC-3 outcomes were also encouraging, demonstrating good compatibility between the THV and the implanted coronary stents. Technical success was 100% with no procedural complications. Device success was 81.0% due to the hemodynamic valve performance (mean gradient > 20 mmHg). Finally, early safety was 90.5% due to the two stroke cases.

Moreover, the largest multicenter study to date comparing snorkel stenting and the BASILICA technique further reinforces the viability of both approaches [7]. This observational registry included 168 high-risk TAVI patients, with 71 undergoing chimney stenting and 97 treated with BASILICA. Patients in the BASILICA group had higher anatomical risk profiles. Despite these differences, both techniques showed similar rates of periprocedural complications, and clinical success exceeded 96% in each group. At one-year follow-up, the incidence of major adverse cardiovascular events was comparable between the chimney (18.7%) and BASILICA (19.9%) groups. Although chimney stenting was associated with a numerically higher cardiovascular mortality rate (6.7% vs. 1.3%), this difference did not reach statistical significance. The cardiovascular-related mortality of our study is in line with the results reported in this study.

As a final remark, snorkel stenting represents a feasible and effective option for patients at high risk of CO. However, several important considerations remain. Concerns persist regarding the long-term implications of a protruding stent in the aortic root, particularly its potential to complicate future transcatheter interventions, both coronary and valvular. Additionally, interactions between the stent and the THV may affect procedural outcomes. Mangieri and colleagues speculated on this possibility, noting a lower incidence of moderate-to-severe paravalvular leak in the BASILICA group, despite a higher use of the Portico valve (Abbott Cardiovascular) in the chimney cohort, a device known to carry an increased risk of paravalvular leak [7], but associated with low CO rates [26].

### 4.1. Medical Therapy

At present, there are no randomized data or formal guideline recommendations specifically addressing the optimal antithrombotic regimen after snorkel/chimney stenting during TAVI. Consequently, management is extrapolated from different areas: antithrombotic therapy after PCI, current recommendations for post-TAVI antithrombotic treatment, and the expert opinions that have reported on snorkel stenting.

According to the 2020 ACC/AHA and the 2021 ESC/EACTS guidelines for TAVI, in patients without another indication for oral anticoagulation, single antiplatelet therapy with aspirin is generally preferred, while dual antiplatelet therapy is no longer routinely recommended, except in selected patients at very low bleeding risk [27,28]. In contrast, PCI guidelines for drug-eluting stents still recommend dual antiplatelet therapy with aspirin and a P2Y12 inhibitor for at least three to six months/1 year, with the duration tailored to bleeding and thrombotic risk [29,30].

In the published literature on snorkel stenting series, most centers have adopted the practice of treating snorkel stents in the same way as coronary drug-eluting stents [7,9,10]. Typically, patients are prescribed dual antiplatelet therapy with aspirin and clopidogrel, followed by lifelong aspirin. In patients who already require oral anticoagulation, for instance, due to atrial fibrillation, the most common practice is to combine the anticoagulant with a single antiplatelet agent, usually clopidogrel, while avoiding triple therapy whenever possible in order to minimize bleeding risk.

### 4.2. Limitations

A key limitation of this study is its retrospective nature, which makes it difficult to fully control for all confounding variables. Moreover, being conducted at a single institution may restrict how well the results translate to other clinical settings. The absence of long-term follow-up also leaves open the possibility that important differences in late survival could emerge over time. Additionally, the relatively small number of patients and the specific characteristics of the study cohort limit the extent to which these findings can be applied to the general population. Moreover, it may have restricted our ability to adequately evaluate the primary outcomes of CO and MI. Notably, the largest study comparing BASILICA with snorkel stenting [7], conducted across nine centers over a six-year period, included 71 patients in the snorkel group. In this context, our single-center experience of 21 patients represents a relatively substantial contribution, considering how rarely this procedure is reported in the literature.

MDCT parameters like VTC, VTA, and VTSTJ were only recently implemented in our center and could not be consistently utilized in this series.

## 5. Conclusions

Snorkel stenting has demonstrated safety and efficacy. However, its broader use is limited by concerns such as stent failure, difficulty with future coronary reaccess, prolonged DAPT requirements, and the risk of stent crush during subsequent valve interventions. A thorough risk assessment should take into account both the presence of a prior bioprosthetic valve and the characteristics of the newly implanted THV.

## Figures and Tables

**Table 1:** Baseline patient characteristics.

Characteristic	*n* = 21
Age, years, mean (SD)	81.3 (6.1)
Female, *n* (%)	16 (76.2)
BSA, m^2^, mean (SD)	1.9 (0.3)
BMI, kg/m^2^, mean (SD)	31.2 (8.5)
NYHA class, *n* (%)	
Class I	1 (4.8)
Class II	6 (28.6)
Class III	10 (47.6)
Class IV	4 (19.0)
BNP, pg/mL, median [IQR]	347 [179–1103]
STS PROM, median [IQR]	5.0 [3.0–8.0]
Hypertension, *n* (%)	20 (95.2)
Dyslipidemia, *n* (%)	20 (95.2)
Diabetes mellitus, *n* (%)	5 (23.8)
Previous endocarditis, *n* (%)	1 (4.8)
Smoke, *n* (%)	
Never	17 (81.0)
Former	4 (19.0)
Chronic lung disease, *n* (%)	3 (14.3)
Creatinine, mg/dL, mean (SD)	1.2 (0.5)
eGFR, mL/min/1.73 m^2^, median [IQR]	53.2 [36.8–70.3]
Dialysis, *n* (%)	0 (0.0)
Previous TIA, *n* (%)	2 (9.5)
Previous stroke, *n* (%)	2 (9.5)
PAD, *n* (%)	2 (9.5)
Previous MI, *n* (%)	3 (14.3)
Previous PCI, *n* (%)	4 (19.0)
Previous CABG, *n* (%)	5 (23.8)
Previous mitral/tricuspid surgery, *n* (%)	0 (0.0)
Atrial fibrillation, *n* (%)	
Paroxysmal	5 (23.8)
Long-standing persistent	1 (4.8)
Permanent	1 (4.8)
Atrial flutter, *n* (%)	3 (14.3)
Preoperative RBBB, *n* (%)	5 (23.8)
Preoperative LBBB, *n* (%)	4 (19.0)
Preoperative pacemaker/ICD, *n* (%)	1 (4.8)

BNP = brain-derived natriuretic peptide; BMI = body mass index; BSA = body surface area; CABG = coronary artery bypass graft; ICD = implantable cardioverter defibrillator; IQR = interquartile range; LBBB = left bundle branch block; MI = myocardial infarction; PAD = peripheral artery disease; RBBB = right bundle branch block; SD = standard deviation; STS PROM = Society of Thoracic Surgeon predicted risk of mortality; TIA = transient ischemic attack.

**Table 2:** Preoperative imaging data.

Parameter	*n* = 21
**MDCT data**	
SoV diameter, mm, mean (SD)	24.6 (6.8)
STJ diameter, mm, mean (SD)	26.1 (4.1)
Mean annulus diameter, mm, mean (SD)	21.2 (2.5)
Max annulus diameter, mm, mean (SD)	23.7 (2.61)
Min annulus diameter, mm, mean (SD)	20.3 (2.3)
Annulus area, mm^2^, mean (SD)	402.0 (59.7)
Annulus perimeter, mm, mean (SD)	71.7 (5.2)
Aortic angle, °degree, mean (SD)	43.4 (10.9)
Left coronary height, mm, mean (SD)	7.9 (3.3)
Right coronary height, mm, mean (SD)	10.6 (5.5)
**Echocardiographic data**	
LVEF, %, mean (SD)	60.0 (7.6)
AVA, cm^2^, median [IQR]	0.90 [0.60–1.66]
iAVA, cm^2^, median [IQR]	0.46 [0.33–0.82]
Peak velocity, m/s, mean (SD)	3.6 (1.0)
Mean gradient, mmHg, mean (SD)	30.0 (15.5–48.5)
Aortic regurgitation, *n* (%)	
None/Trace	9 (42.9)
Mild	1 (4.8)
Moderate	2 (9.5)
Severe	9 (42.9)
**Previous bioprostheses**	
Degenerated bioprosthesis, *n* (%)	18 (85.7)
Trifecta	7 (33.3)
Mitroflow	3 (14.3)
Freestyle	2 (9.5)
CoreValve	2 (9.5)
Evolut R	1 (4.8)
Evolut PRO	1 (4.8)
Perceval	1 (4.8)
Toronto Bilinks	1 (4.8)
Degenerated bioprosthesis size, *n* (%)	
19	1 (4.8)
21	7 (33.3)
23	4 (19.0)
25	1 (4.8)
29	5 (23.8)

AVA = aortic valve area; iAVA = indexed aortic valve area; IQR = interquartile range; LVEF = left ventricular ejection fraction; MDCT = multidetector computed tomography; SD = standard deviation; SoV = sinus of Valsalva; STJ = sinotubular junction.

**Table 3:** Perioperative outcomes.

Perioperative Outcomes	*n* = 21
Transfemoral approach, *n* (%)	20 (95.2)
Transaxillary approach, *n* (%)	1 (4.8)
Pre-dilatation, *n* (%)	3 (14.3)
Post-dilatation, *n* (%)	7 (33.3)
Valve-in-Valve, *n* (%)	18 (85.7)
Implanted Valve	
Evolut R	2 (9.5)
Evolut PRO	2 (9.5)
Evolut PRO+	5 (23.8)
Evolut FX	4 (19.0)
Sapien 3	2 (9.5)
Sapien Ultra	6 (28.6)
Device size, *n* (%)	
20	1 (4.8)
23	14 (66.7)
26	5 (23.8)
34	1 (4.8)
Snorkel coronary, *n* (%)	
LMC	9 (42.9)
RCA	10 (47.6)
LMC + RCA	2 (9.5)
Surgical conversion, *n* (%)	0 (0.0)
MCS, *n* (%)	0 (0.0)
Intraoperative transfusions, *n* (%)	0 (0.0)
Valve mispositioning, *n* (%)	0 (0.0)
Second valve deployment, *n* (%)	0 (0.0)
Annulus rupture, *n* (%)	0 (0.0)
Coronary obstruction, *n* (%)	0 (0.0)
Major vascular complication, *n* (%)	0 (0.0)
Minor vascular complication, *n* (%)	0 (0.0)
Stroke	1 (4.8)
Disabling	1 (4.8)
Postoperative MI	0 (0.0)
New LBBB, *n* (%)	4 (19.0)
New pacemaker implant, *n* (%)	1 (4.8)
Postoperative transfusion, *n* (%)	1 (4.8)
Length of stay, days, median [IQR]	1 [1,2]
30-day mortality, *n* (%)	0 (0.0)
30-day CV readmission, *n* (%)	0 (0.0)
**Echocardiographic data**	
LVEF, %, mean (SD)	60.9 (9.2)
EOA, cm^2^, mean (SD)	1.66 (0.55)
iEOA, cm^2^, mean (SD)	0.87 (0.34)
Peak velocity, m/s, mean (SD)	2.6 (0.5)
Mean gradient, mmHg, mean (SD)	16.1 (7.7)
Paravalvular leak, *n* (%)	
None/Trace	19 (90.5)
Mild	2 (9.5)
Central leak, *n* (%)	
None/Trace	21 (100)
Mild	0 (0.0)

CVA = cerebrovascular accident; i/EOA = indexed/effective orifice area; IQR = interquartile range; LBBB = left bundle branch block; LMC = left main coronary; LVEF = left ventricular ejection fraction; MCS = mechanical circulatory support; MI = myocardial infarction; RCA = right coronary artery; SD = standard deviation.

**Table 4:** VARC-3 and follow-up outcomes.

VARC-3 and Follow-Up Outcomes	*n* = 21
**VARC-3 outcomes**	
Technical success, *n* (%)	21 (100)
Device success, *n* (%)	17 (81.0)
Early safety, *n* (%)	19 (90.5)
**Clinical outcomes**	
Follow-up, years, median [IQR]	1.25 [1.05–3.05]
Rehospitalization for heart failure, *n* (%)	5 (23.8)
Aortic valve reintervention, *n* (%)	0 (0.0)
Overall survival, *n* (%)	17 (81.0)
CV-related death, *n* (%)	2 (9.5)
Total new pacemaker, *n* (%)	2 (9.5)
Stroke, *n* (%)	1 (4.8)
Disabling	1 (4.8)
Endocarditis, *n* (%)	1 (4.8)
Myocardial infarction, *n* (%)	1 (4.8)
**1-month echocardiographic data (*n* = 19)**	
LVEF, %, mean (SD)	61.4 (8.5)
EOA, cm^2^, mean (SD)	1.54 (0.50)
iEOA, cm^2^, mean (SD)	0.80 (0.27)
Mean gradient, mmHg, median [IQR]	17.0 [12–19]
Aortic regurgitation, *n* (%)	
None/Trace	16 (84.2)
Mild	3 (15.8)
**1-year echocardiographic data (*n* = 13)**	
LVEF, %, mean (SD)	68.9 (7.4)
EOA, cm^2^, mean (SD)	1.65 (0.62)
iEOA, cm^2^, mean (SD)	0.83 (0.25)
Mean gradient, mmHg, median [IQR]	13.0 [7.0–26.0]
Aortic regurgitation, *n* (%)	
None/Trace	10 (76.9)
Mild	3 (23.1)

CV = cardiovascular; i/EOA = indexed/effective orifice area; LVEF = left ventricular ejection fraction; SD = standard deviation.

## Data Availability

Data supporting the conclusions of the study can be made available by the corresponding author upon reasonable request, pending institutional approval.

## Abbreviations

The following abbreviations are used in this manuscript:

CO	Coronary obstruction
MI	Myocardial infarction
TAVI	Transcatheter aortic valve implantation
THV	Transcatheter heart valve
